# Mr. Chamberlain on Friendly Workers

**Published:** 1893-01-21

**Authors:** 


### The Hospital

An Institutional Journal of

Science, Medicine, Nursing, and Philanthropy.

*For Hospitals, Asylums, Infirmaries, Dispensaries, Medical Practitioners, Students, Nurses, and the Charitable Public.*

Vol. XIII.—No. 330.] [Jan. 21, 1893.

### Mr. Chamberlain on Friendly Workers.

Mr. Joseph Chamberlain, presiding on Monday at the second annual meeting of the West Birmingham Relief Fund, took occasion not only to recount the success of a new experiment in poor relief, but also to expound the general principles upon which success in such cases must always depend. By this time everybody knows that Mr. Chamberlain is an excellent man of business. In commerce, in local, and later in Imperial politics he has been pre-eminently successful, and his success has in all these instances depended upon a clear head and a resolute will. It is a great advantage to the cause of poor relief generally that Mr. Chamberlain has made a public utterance upon the subject. He is, as we have said, one of our most distinguished specialists in practical affairs, and wise philanthropists will give to his exposition of principles the careful thought which experience, combined with capacity, is entitled to look for.

The West Birmingham Relief Fund has been in operation nearly two years, and it has been a conspicuous success. That was Mr. Chamberlain's text. What have been the causes of its success? To the elucidation of this question the speaker devoted his brief sermon. Like his former leader in the House of Commons, Mr. Chamberlain insisted upon three cardinal points, and those three points undoubtedly indicated the true solution of the great problem. First of all, Mr. Chamberlain says, your organization for the relief of distress must be "permanent." Secondly, it must be "local"; and thirdly, it must be carried out by "voluntary" workers.

When Mr. Chamberlain insists that any organisation for the effectual relief of the poor must be "permanent" he gives utterance to a proposition which is self-evident to all practical workers in this particular philanthropic field. Funds which are raised by sudden impulses and distributed by sudden impulses are now generally discredited; and that for the sufficient reason that they have almost invariably been found in practice to do vastly more harm than good. A permanent fund is like a preventive measure in the art of sanitation. "An ounce of prevention is worth a pound of cure" is our sanitary proverb. Or it may be likened to a garden well kept by skilful gardeners. Its distributors always see, as the gardener sees small weeds, the causes of suffering and distress among the poor in their earliest and smallest stages; and they can, in almost every case effectually nip them in the bud. There is often as much difference between distress taken in its beginnings and distress allowed to become universal as there is between the nipping of a spring bud and the cutting down of a forest of oaks. The one great advantage of a "permanent" as against what we may call an "impulsive" fund is that the distributors of it, being permanently in office, get to know all the facts and all the circumstances of the daily life of the recipients. They know the industrious, and they know the idle: they know the shy and the proud, and they know the bold and the shameless; they know the occupations of the toilers, and they know the masters who provide them with employment. They have, in short, the very first and most fundamental of all the conditions of successful work, a full and intelligent knowledge and comprehension of all the facts.

The second condition of effectually, and in a generous spirit, relieving the industrious poor is that the organization must be "local." As Mr. Chamberlain convincingly says: You can only be successful in the fullest measure "by enlisting local interest, local knowledge, and local pride. A man is more inclined to relieve his own neighbours, friends, and fellow-workers than he is to help entire strangers." These are the very tones of the neighbourly philanthropist who takes a practical and human view of the situation. Philanthropy on a large scale is like Imperial Government on a large scale—a very difficult task. Local self-government is the soul of effective charity, as it is of all State policy where the State is of vast proportions. "You cannot," says Mr. Chamberlain, "have a central organisation for dealing with the whole of the poverty of Birmingham. There must be applications that demand personal supervision, and if there be but one actual organisation there must often be injurious delay," and not merely delay, the speaker might have added, but a very much lower degree of practical efficiency all round. Mr. Chamberlain concluded his argument on this point by expressing the hope that he would live "to see the time when every division and every ward in Birmingham would have a separate relief organisation."

It will surprise some persons, whom we need not name, to be told by Mr. Chamberlain that "the third and most important feature of all" in the new West Birmingham Relief Fund is "that the association relies upon voluntary work," and that, consequently, "there are no administrative expenses." On this most fundamental question of "voluntary" as against "paid" workers, Mr. Chamberlain was convinced and convincing. "The advantages," he insisted, "were very clear." "In the first place there is a much better selection of cases. There are many deserving people who have a perfect horror of telling their circumstances to paid officials; but they will tell them freely to their neighbours and friends;" and even when shame keeps them silent, "their neighbours have generally a very good idea of what is going on" in the family.

It is a circumstance which deserves special mention, that of the voluntary workers who have taken up the Relief Fund in West Birmingham no less than three-fourths are working men. When it is stated that the total sum expended during the year under consideration amounted to less than two hundred pounds, it is obvious that the British workman, even when spending other people's money, is not inclined to be too sentimentally generous to his fellow-workman in distress. Mr. Chamberlain was well entitled to congratulate his audience on this peculiar and interesting fact: "Three-fourths of their visitors," he said, "were working men, who gave up their spare time to help their fellow-workers. None of them knew for certain that misfortune might not dog their own footsteps. They were, therefore, the careful trustees of the fund which they administered, and took great care that the money was not wasted."

We cannot help comparing this exposition of principles, and record of successful working, with the principles upon which the "North Kensington Friendly Workers' Association" is based, and the success of its three years' operations. The North Kensington Association has been somewhat longer in existence, but the principles upon which the two societies are founded are in many respects similar, and indeed, up to a certain point, identical. The North Kensington Association aims at being "permanent," and so does the Birmingham Fund. Similarly both the schemes insist upon the necessity of being "local," and both pin their faith to "voluntaryism," and "voluntary workers." The North Kensington scheme has an advantage in that it has succeeded in uniting philanthropists of all classes and religious denominations under its single banner. This is a great step in advance. On the other hand, Mr. Chamberlain, in enlisting so large a proportion as two-thirds of working men for his voluntary workers, has almost worked a miracle.

The conclusion of the whole matter would seem to be that in some such scheme as the West Birmingham Fund or the North Kensington Friendly Workers' Association lies the real answer to the question of what is to be done in "hard times" for the help of the industrious and deserving poor. Those who, for any reason, are past all hope of recovering their industrial and social position must perforce be left to the Poor Law. But those who, by a little timely and friendly help, can be set upon their legs again, after a period of unlooked-for misfortune, should have that help extended to them. On this point everybody is agreed. The practical question is—How is it to be done? And the answer seems undoubtedly to be—By permanency of organisation, localisation, voluntary work, and last, but not least, intelligent co-operation.

